# Retinitis Pigmentosa Sine Pigmento in a Carrier of Usher Syndrome

**DOI:** 10.7759/cureus.37719

**Published:** 2023-04-17

**Authors:** Sofía C Ayala Rodríguez, Estefania Ramirez Marquez, Andrea Robles Bocanegra, Natalio Izquierdo, Armando L Oliver

**Affiliations:** 1 Ophthalmology, University of Puerto Rico School of Medicine, Medical Sciences Campus, San Juan, PRI; 2 Ophthalmology, San Juan Bautista School of Medicine, Caguas, PRI; 3 Surgery, University of Puerto Rico School of Medicine, Medical Sciences Campus, San Juan, PRI

**Keywords:** case report, deaf-blindness, sensorineural hearing loss, retinitis pigmentosa sine pigmento, usher syndrome

## Abstract

We report a carrier of Usher syndrome type I with retinitis pigmentosa sine pigmento. A 71-year-old male was referred for further evaluation of severe, progressive, painless vision loss in both eyes over the course of four years. He had bilateral sensorineural hearing loss. Upon a comprehensive examination, his best-corrected visual acuity was 20/100 in the right eye and 20/40 in the left eye. He had an unremarkable anterior segment examination and normal intraocular pressures in both eyes. Upon fundus examination, the patient had pale discs, optic disc cupping, and multiple scattered drusen in the macula and at the midperiphery of both eyes. Optical coherence tomography showed retinal nerve fiber layer thinning in all quadrants. The visual field was severely constricted in both eyes. A comprehensive workup for infectious and inflammatory causes, as well as a brain MRI, was unremarkable. Sequencing analysis showed that he carried a heterozygous pathogenic mutation, USH1C c.672C>A (p.Cys224*) variant. Usher syndrome is a rare genetic disease characterized by hearing loss and retinitis pigmentosa. Our case suggests that patients and carriers of Usher syndrome may have a phenotype compatible with retinitis pigmentosa sine pigmento.

## Introduction

Usher syndrome (USH) is a rare, autosomal recessive disorder characterized by progressive retinal dystrophy, sensorineural hearing loss (SNHL), and variable vestibular dysfunction [1]. This syndrome is responsible for about half of all hereditary deaf-blindness cases [2]. Similar to other autosomal recessive disorders, USH incidence increases with consanguinity [1].

A common clinical manifestation of the disease in patients with retinitis pigmentosa (RP) is progressive vision loss resulting from the degeneration of retinal photoreceptors [3]. Symptoms include nyctalopia, decreased visual acuity and contrast sensitivity, peripheral vision restriction, and end-stage central vision loss [3,4]. Upon fundus examination, there is optic disc atrophy, vessel attenuation, and mid-peripheral bony spicules [3,5]. Usher syndrome can be classified into three clinical types according to the severity and progression of SNHL and age at the onset of RP [1]. Currently, there is no cure for this syndrome; however, a timely diagnosis and early intervention of Usher syndrome are essential for adequate rehabilitation of potential developmental interferences.

Kwiecień and co-workers first reported a patient with Usher syndrome presenting as retinitis pigmentosa sine pigmento [4]. Hereby, we report the phenotype of a carrier of Usher syndrome type I with an exon 8, c.672C>A (p.Cys224*) variant.

## Case presentation

A 71-year-old male patient had bilateral SNHL during adulthood and reported a four-year history of severe, progressive, painless, vision loss in his right eye (OD). His family history was remarkable for deafness in his mother, maternal uncle, and brother. Past medical and surgical history was remarkable for rheumatoid arthritis, hypertension, and pterygium surgery of the left eye (OS).

**Figure 1:**
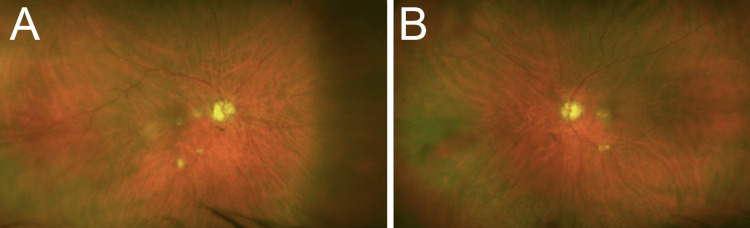
Ultra-widefield fundus images of the patient. The images show pale discs with disc cupping and multiple scattered drusen in the macula and at the midperiphery of the right (A) and left (B) eye.

Upon comprehensive ophthalmic examination, his best-corrected visual acuity was 20/100 OD and 20/40 OS with a manifest refraction of −0.50 +2.00 × 165 OD and -0.50 +1.25 × 010 OS. Intraocular pressure was 21 mmHg in both eyes (OU). He was unable to do the Ishihara color plates test. Extraocular movements were within normal limits, without nystagmus. Pupils were round and reactive to light, without any relative afferent defect. Anterior segment examination was unremarkable. Upon slit lamp examination, the patient had nuclear sclerosis 2+ of both lenses. Upon fundus examination, the patient had pale discs with optic disc cupping (C/D ratio of the OD: horizontal: 0.6, vertical: 0.7; C/D ratio of the OS: horizontal: 0.8, vertical: 0.7), and multiple small drusen in the periphery of the macula bilaterally (Figure 1). Retinal midperiphery had no pigment. Optical coherence tomography (OCT) of the disks showed moderate disk cupping OU with retinal nerve fiber layer thinning in all quadrants of the OD, and in the temporal, superior, and inferior quadrants of the OS, while the macular OCT revealed a significantly decreased cube average thickness and volume OU. There was no evidence of intraretinal cystic macular edema, macular thickening, or subretinal fluid OU. Electroretinogram (ERG) showed decreased amplitude in both scotopic and photopic phases.

Upon visual field examination, the patient had ring scotomas OU with decreased mean deviation (-27.16 dB and -27.57 dB in the OD and OS, respectively) and increased pattern standard deviation results (7.14 dB and 6.51 dB in the OD and OS, respectively).

Upon comprehensive systemic workup, there was no evidence of infectious, inflammatory, or other systemic diseases. Upon genetic analysis using an Invitae IRD panel (Invitae Corporation, San Francisco, CA), the patient had a heterozygous pathogenic mutation, c.672C>A (p.Cys224*) variant in the USH1C gene.

## Discussion

Usher syndrome is an autosomal recessive syndromic ciliopathy that is clinically and genetically heterozygous [1]. It is characterized by RP, SNHL, and in some cases, vestibular dysfunction. The most common and severe form of this syndrome is type I, also known as USH1, and is characterized by severe-to-profound SNHL, RP, and vestibular dysfunction with onset before puberty [1]. Fundus examination, ERG, visual field, physical evaluation, and family history help guide the diagnosis of this disease; however, genetic testing is the most definitive diagnostic tool. To this day, there are six causative genes identified underlying Usher subtype 1, one of which is the USH1C [1].

Our patient had a history of bilateral SNHL and severe, progressive, painless vision loss OD, as well as a family history of deafness. These characteristics did not deviate from those found in classic cases of Usher syndrome. The patient included in our study is classified as a carrier of an atypical form of RP, referred to by several authors as sine pigmento, because of the absence of peripheral bone-spicule-like pigmentation on the examination of the retina [5,6]. It is our impression that patients and carriers of Usher syndrome may present RP in the absence of peripheral bone-spicule-like pigmentation. The carrier status of our patient is a possible cause for his late onset of phenotypic presentation.

Furthermore, our patient carries a heterozygous pathogenic mutation on the USH1C gene with an exon 8, c.672C>A (p.Cys224*) variant. This mutation creates a premature translational stop signal in the USH1C gene leading to the potential absence or disruption of protein function [7]. The USH1 gene encodes harmonin, a protein required for the development and maintenance of mechanotransduction in cochlear hair cells; thus, making USH an inherited ciliopathy [8]. Unlike most USH1 patients, our patient reported normal vestibular function in infancy and gradual development of SNHL in adulthood, which might be related to his carrier status.

Identification of gene expression phenotypes in carriers of Usher syndrome is crucial to enhance and facilitate the study of carriers, who are more prevalent than those with the genetic disease. Using timely genetic analysis and diagnosis may grant patients with Usher syndrome the opportunity to identify offspring's risk of recurrence and receive early education, rehabilitation, counseling, and support. Further studies should be conducted to compare the phenotypic variability between control patients and carriers.

## Conclusions

Usher syndrome is a rare genetic disease characterized by hearing loss and the classical features of RP. However, our case suggests that patients and carriers of Usher syndrome may also have RP sine pigmento. Patients with early onset of SNHL and RP should be genetically tested to diagnose and provide comprehensive care accordingly in the early stages of child development.
